# Nursing Science Interventions in Aging

**DOI:** 10.1093/geroni/igac062

**Published:** 2022-11-26

**Authors:** Meghan K Mattos, Jennifer H Lingler

**Affiliations:** Acute and Specialty Care Department, University of Virginia School of Nursing, Charlottesville, Virginia, USA; Department of Health and Community Systems, School of Nursing, University of Pittsburgh, Pittsburgh, Pennsylvania, USA

What is “nursing science,” and why do we acknowledge this subfield as a distinct entity within the broad umbrella of “science?” Nursing science is recognized as both basic science (Fawcett, 2020) that focuses on nursing discipline-specific knowledge and applied science. Bottorff (1991) defined nursing science as the “branch or body of knowledge that is characteristically different from the knowledge that is aimed at and achieved by other discipline.” However, when considering nursing science in this context, nursing practice requires knowledge outside of “nursing science,” which is not specific to nursing, such as psychology. The complexity of thinking of nursing science as a basic science and applied science presents challenges beginning at the level of education and extending through the development and implementation of policy. Nurse education is rich in content, ranging from pharmacology to pathophysiology to nursing care across the life span, including the care of older adults. The National Institute of Nursing Research (NINR) 2022–2026 Strategic Plan focuses on how nurse scientists’ research lenses are rooted in nursing’s expertise in biological, behavioral, social, and public health sciences and long-standing values of social justice, holism, and collaboration (National Institute of Nursing Research, 2022). Theory also plays a significant role in nursing science, wherein research is typically designed using middle-range or situation-specific theories. Nursing science aims to “expand knowledge about human experiences through research and creative conceptualization” (Fawcett, 2001). Nursing research occurs from the bench to the bedside, uniquely positioning nurses to translate their work, typically inter- or multidisciplinary endeavors, and inform practice and policy.

This special issue of *Innovation in Aging* is an opportunity to highlight cutting-edge nursing science research that addresses challenges in the setting of aging and life-span health. NINR’s three goals for nursing science guide the content of this special issue: (a) dismantling structures that perpetuate racism and impede health equity, (b) multilevel perspective to develop and implement interventions to address social determinants of health, and (c) holistic approaches to advance precision health and health care. Nursing research is not limited to a particular disease or symptom, and it is rather the holistic and patient-centered approach to care that gives nurses the unique lens to engage in nursing science. As the largest health profession in the United States and a critical component of the global health care workforce, nurse facilitation and representation in team science is critical to innovation and the development of interventions to improve the health and quality of life of all. Nurses are essential members of these teams, and this *Innovation in Aging* special issue presents an opportunity to highlight nursing intervention development, testing, and implementation. All but one article in this special issue is written by a registered nurse and/or nurse scientist. As expected for an issue examining nursing interventions in older adults, funding for the work was supported by NINR and National Institute on Aging (NIA) of the National Institutes of Health (NIH). NIA-funded studies focused on dementia populations, and there was diversity across studies’ target study populations funded by NINR and other non-NIH funding agencies.

This special issue was driven by NINR’s preview of the 2022–2026 Strategic Plan, and the articles presented in this issue focus on two areas:

(1) Holistic approaches to advance precision health and health care
(2) Multilevel perspectives to develop and implement interventions to address social determinants of health

## Holistic Approaches to Advance Precision Health and Health Care

Nursing science contributes to precision health and has been addressed through a long-standing history of targeted work focused on symptom science. In 2019, Fu et al. presented the importance of nursing science in the implementation of precision health and how nurse leaders are well-positioned due to “interprofessional collaboration, community outreach efforts, and coordination of care” (Fu et al., 2019). This special issue presents articles using technology to promote precision health and one focused on application of an evidence-based framework. Coleman et al. (2022) describe the process of developing and testing an online version of communication training to reduce elderspeak and improve behavioral symptoms of nursing home residents with dementia. Site-specific implementation strategies and approaches to successful implementation and process evaluation are described. Happ (2022) provides commentary on the considerations when implementing a complex intervention and implementation science. Ge et al. (2022) describe the Engaging with Aging framework to help understand the daily living process for older adults who are managing age-related changes (ARCs). The study supports use of the framework to understand how and why older adults use adaptative behaviors to address ARCs and may be used to help guide precision medicine in the future. Liu et al. (2022) present findings from 160 videotaped mealtime observations of nursing home staff and residents across nine nursing homes. This comprehensive descriptive study found that person-centered approaches and residents’ challenging behaviors were associated with food intake and also that facilitation of positive staff–resident dyadic interactions may be crucial to the promotion of adequate resident food intake. Leutwyler et al. (2022) present an innovative approach to improving walking speed in older adults with serious mental illness. Over 10 weeks, participants engaged in group sessions playing active video games on an Xbox 360. Using this novel and innovative approach to engaging in physical activity, the authors found statistically and clinically significant improvements in walking speed that may potentially impact long-term health outcomes. LeBaron (2022) presents challenges and opportunities for designing and employing remote health monitoring technologies in older adults with cancer. Poised at the intersection of direct patient care and user-centered design, nurses are uniquely equipped to leverage their experiences and expertise. Specific focal topics, such as addressing health equity and research-related considerations, are highlighted to provide a roadmap for early-stage and established researchers. Opportunities for nurses to lead efforts in holistic approaches to the advance precision health and health care are vast.

## Multilevel Perspectives to Develop and Implement Interventions to Address Social Determinants of Health

Social determinants of health, including “conditions in which people are born, live, learn, work, play, and age,” are one of the five research lenses presented by NINR to help identify approaches to improve health and quality of life. Across four articles featured in this issue, nursing science uses multilevel methods and approaches to address social determinants of health to improve patient outcomes. To understand antipsychotic medication use among assisted living/residential care (AL/RC) residents, Dys and Carder (2022) conducted semistructured interviews with AL/RC staff. Surprisingly, authors found that staff’s perceived level of agency decreased when care or services were closer to the patient level. For example, direct providers, such as nurses, were less likely to have a strong sense of agency compared with participants who were not involved in daily interactions within the AL/RC in as-needed antipsychotic medication use. Montano et al. (2022) created an innovative academic training program for health care students and faculty to promote care of older, community-dwelling adults. Interdisciplinary perspectives are presented from pharmacy, therapy, nursing, social work, medicine, and public health students; community-dwelling older adults; and primary care physicians. Program evaluation by students focused on the unique opportunities to learn about care of older adults outside of office visits. Authors found that training programs promoting interprofessional education provide a comprehensive approach to prevention and health promotion while also addressing social determinants of health by meeting people “where they are.” Kiyoshi-Teo et al. (2022) present findings from a survey study that examined the impact of COVID on changes in fall risk and day-to-day activities of assisted living residents. Findings support NINR’s strategic plan to identify and address multilevel factors to optimize health. Sullivan et al. (2022) provide a compelling presentation of critical quality-of-life and social determinants of health factors that influence the use of hospice services in dementia caregiving dyads. Using novel machine learning approaches, the authors identify opportunities to tackle structural barriers and promote precision health.

## Next Steps

Nursing science is a distinctive entity within the broad umbrella of science and, taken together, the articles contained in this special issue demonstrate the value of raising and addressing research questions of relevance to the care of older adults from the unique lens of a nurse scientist. Much of this innovative work is exploratory or preliminary in nature. Rigorous and reproducible confirmatory investigations and effectiveness trials, especially with racially and ethnically representative samples, are needed to grow the evidence base for testing the models, and translating into clinical practice the interventions, showcased in this special issue. Curating this celebration of the promise of gerontological nursing science has been a privilege. We extend our most heartfelt thanks to Steven Albert, editor-in-chief, and Karen Jung, managing editor, for entrusting us with this opportunity and shepherding us through this effort.
